# Adjuvant chemotherapy after surgery can improve clinical outcomes for patients with IB2-IIB cervical cancer with neoadjuvant chemotherapy followed by radical surgery

**DOI:** 10.1038/s41598-018-24413-z

**Published:** 2018-04-24

**Authors:** Haiying Sun, Kecheng Huang, Fangxu Tang, Xiong Li, Xiaoli Wang, Sixiang Long, Shasha Zhou, Suolangquzhen, Jianwei Zhang, Ruoqi Ning, Shuang Li, Shixuan Wang, Ding Ma

**Affiliations:** 1 Department of Obstetrics and Gynecology, Tongji Hospital, Tongji Medical College, Huazhong University of Science and Technology, Wuhan, P.R. China; 2 Cancer Biology Research Center, Tongji Hospital, Tongji Medical College, Huazhong University of Science and Technology, 1095 Jiefang Ave, 430030 Wuhan, Hubei P.R. China

## Abstract

The aim of the study is to evaluate the efficacy of postoperative treatments based on pathological response for cervical cancer patients who received neoadjuvant chemotherapy (NACT) followed by radical surgery. Firstly, a total of 756 cervical squamous cell cancer (SCC) patients with FIGO IB2-IIB were included in this retrospective study. Then data from a prospective cohort of 393 patients was employed for further validation. Overall survival (OS) and disease-free survival (DFS) were assessed. In the retrospective study, SCC patients who accepted adjuvant chemotherapy after radical surgery had a relatively better OS than those who received no therapy (P = 0.08, HR = 0.57). The result was more noticeable in the prospective cohort study (P = 0.006, HR = 0.28). In the combined analysis, adjuvant chemotherapy improved clinical outcomes compared with no therapy (P = 0.002 and 0.04 for OS and DFS). Particularly for patients with extra-cervical residual disease, adjuvant chemotherapy improved OS (log-rank P = 0.008, 0.004 and 0.001 in the retrospective, prospective and combined studies). Optimal response patients had good outcomes even without therapy. Our study indicates that adjuvant chemotherapy can benefit clinical outcomes for SCC patients with NACT followed by radical surgery, especially those with extra-cervical residual disease. For optimal response patients, there may be no need for further treatment. This finding needs to be validated in more future studies.

## Introduction

Cervical cancer is the third most frequent female malignancy worldwide. Approximately 500,000 new cases of cervical cancer occur each year, with 80% of these being in developing countries^1^. In recent years, NACT followed by radical surgery has emerged as a new option for treating cervical cancer^2–4^. In China, NACT followed by radical surgery was used for several years in patients with FIGO stage IB2-IIB cervical cancer^3^.

To improve long-term survival and decrease recurrence, researchers have evaluated postoperative treatment after radical surgery for cervical cancer patients^5–10^. Conversely, few data are available on adjuvant postoperative therapy in patients treated with NACT and radical hysterectomy. Angioli R *et al*.^11^ evaluated the efficacy of adjuvant chemotherapy for cervical cancer patients with NACT followed by radical surgery. All enrolled patients received 3 cycles of platinum-based chemotherapy every 3 weeks. They concluded that the adjuvant chemotherapy regimen after neoadjuvant chemotherapy and radical surgery represented a valid treatment for patients with locally advanced cervical cancer. Huali Wang *et al*.^12^ found that adjuvant radiotherapy and chemotherapy effectively decreased the recurrence rate for patients who accepted NACT followed by radical surgery. Maki Matsumura *et al*.^12^ found that NACT followed by surgery with postoperative chemotherapy but without radiotherapy offered a viable option for treating stage IB2-IIB cervical cancer.

Several studies indicated that the pathological response seen in surgical specimens strongly predicts survival for patients with NACT followed by radical hysterectomy^13–15^. However, few data exist on the options and efficacy of postoperative treatments based on the pathological response of the surgical specimens. To date, we have only found one study on this topic. Landoni F. *et al*.^14^ indicated that optimal responders for FIGO stage IB2-IIB cervical cancer after chemo-surgical treatment do not need further treatment, and adjuvant chemotherapy after surgery could benefit patients with suboptimal response and intra-cervical residual disease. However, adjuvant treatments did not appear to benefit the clinical outcomes of patients with extra-cervical residual disease compared with those who received no further treatment. However, no related data exist on postoperative treatment based on the pathological response for SCC patients in China. Thus, we designed this study to investigate the effectively of pathological response and postoperative treatments of IB2-IIB SCC patients who received NACT followed by radical surgery.

## Results

### Patient characteristics

In the retrospective analysis, we included 756 SCC patients with stage IB2-IIB who received neoadjuvant chemotherapy followed by radical hysterectomy (Table 1). The median age was 44 years (range 39–50). Of the 756 patients, 58 experienced optimal response. Intra-cervical and extra-cervical residual disease occurred in 363 and 163 patients, respectively, and 172 had no response. Post-surgery, 307, 299 and 144 patients received no further treatment, adjuvant chemotherapy and adjuvant radiotherapy (including concurrent chemo-radiotherapy), respectively.

**Table 1:** Clinical characteristics of all patients.

Characteristics	Retrospective study (n = 756)			Prospective cohort study (n = 393)	
	No.	%		No.	%
**Age (25th–75th percentiles) (year)**					
Median	44			45	
Range	39–50			40–49	
**Tumor size (25th–75th percentiles) (cm)**					
Median	4.0			4.0	
Range	3.5–5.0			3.0–5.0	
**Tumor grade**					
G1	51		6.7	33	8.4
G2	326		43.1	196	49.9
G3	210		27.8	135	34.4
Undetermined	169		22.4	29	7.4
**FIGO stage**					
IB2	184		24.3	104	26.5
IIA	240		31.7	100	25.4
IIB	332		43.9	189	48.1
**Pathological response**					
Optimal response	58		7.7	41	10.4
Intra-cervical residual disease	363		48.0	222	56.5
Extra-cervical residual disease	163		21.6	60	15.3
No response	172		22.8	70	17.8
**Postoperative treatment**					
No further treatment	307		40.6	83	21.1
Chemotherapy	299		39.6	257	65.4
Radiotherapy	144		19.0	52	13.2
Unknown	6		0.7	1	0.2

FIGO, International Federation of Gynecology and Obstetrics.

Among the total 567 cervical cancer patients in the prospective cohort, 393 SCC patients were included. All underwent neo-adjuvant platinum-based chemotherapy and radical surgery (Supplementary Figure 1). The mean age was 45 years (40–49). Of the 393 patients, 83, 257 and 52 patients received no further treatment, adjuvant chemotherapy and adjuvant radiotherapy, respectively. The details are shown in Table 1.

### Cox analysis for post-surgery treatments and survival

Based on univariate Cox regression analysis in the retrospective study, patients who accepted adjuvant chemotherapy had a relatively better OS than those who did not receive therapy (P = 0.08, 0.57 [0.31, 1.08]). This result was more noticeable in the prospective study (P = 0.006, 0.28 [0.12, 0.69]). In addition, patients with chemotherapy after surgery had a lower recurrence rate than those without therapy in the prospective study (P = 0.04, 0.44 [0.2, 0.95]). Multivariate Cox regression analysis revealed that patients had better survival rates with adjuvant chemotherapy after surgery than those without therapy, particularly OS, in the prospective study (P = 0.02, 0.39 [0.17, 0.86] and P = 0.002, 0.22 [0.088, 0.57] for DFS and OS). Combined analysis revealed that adjuvant chemotherapy played a role in OS and DFS compared with no therapy (P = 0.04, 0.002 in the univariate Cox regression, and P = 0.02 and 0.001 in the multivariate Cox regression analyses for DFS and OS) (Supplementary Tables).

**Figure 1:**
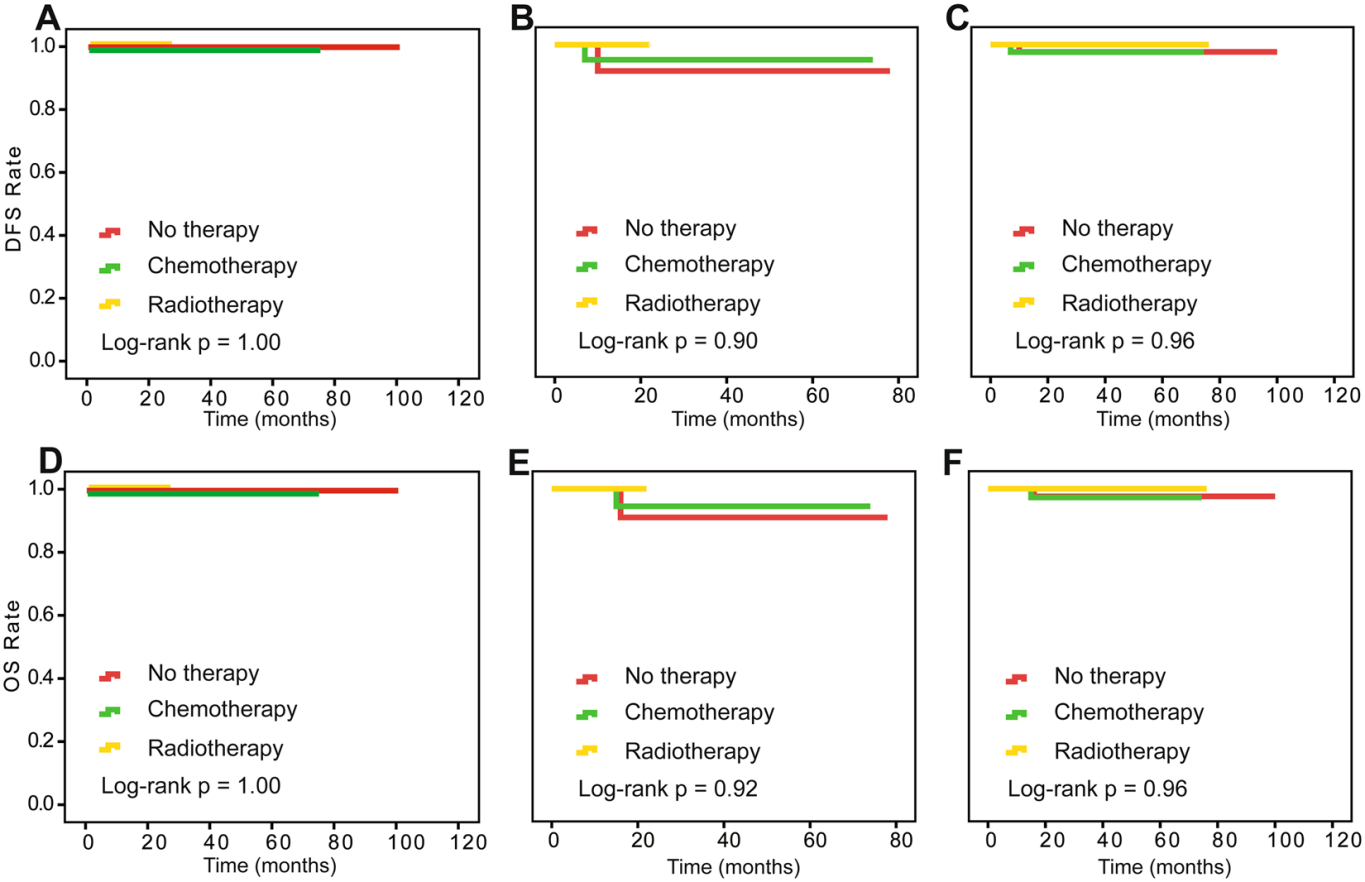
Kaplan-Meier survival estimates for optimal-response patients with cervical cancer from the retrospective study, the prospective cohort and the two studies combined. Disease-free survival (DFS) curves of evaluated patients in the retrospective study (**A**), the prospective cohort (**B**) and the combined results (**C**). Overall survival (OS) curves of evaluated patients in the retrospective study (**D**), the prospective cohort (**E**) and the combined results (**F**).

**Figure 2:**
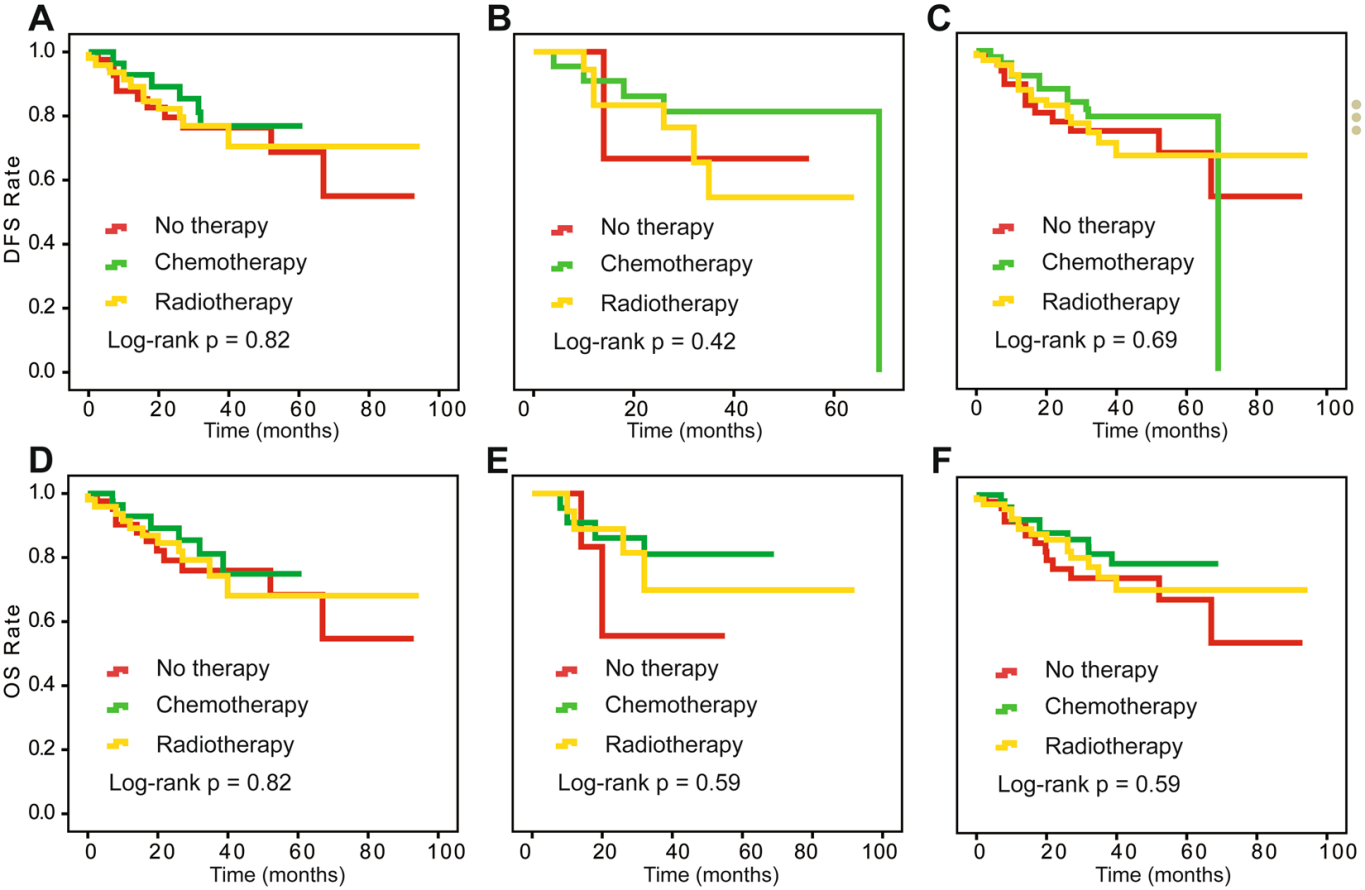
Kaplan-Meier survival estimates for patients with intra-cervical residual disease from the retrospective study, the prospective cohort and the two studies combined. Disease-free survival (DFS) curves of evaluated patients in the retrospective study (**A**), the prospective cohort (**B**) and the combined results (**C**). Overall survival (OS) curves of evaluated patients in the retrospective study (**D**), the prospective cohort (**E**) and the combined results (**F**).

**Figure 3:**
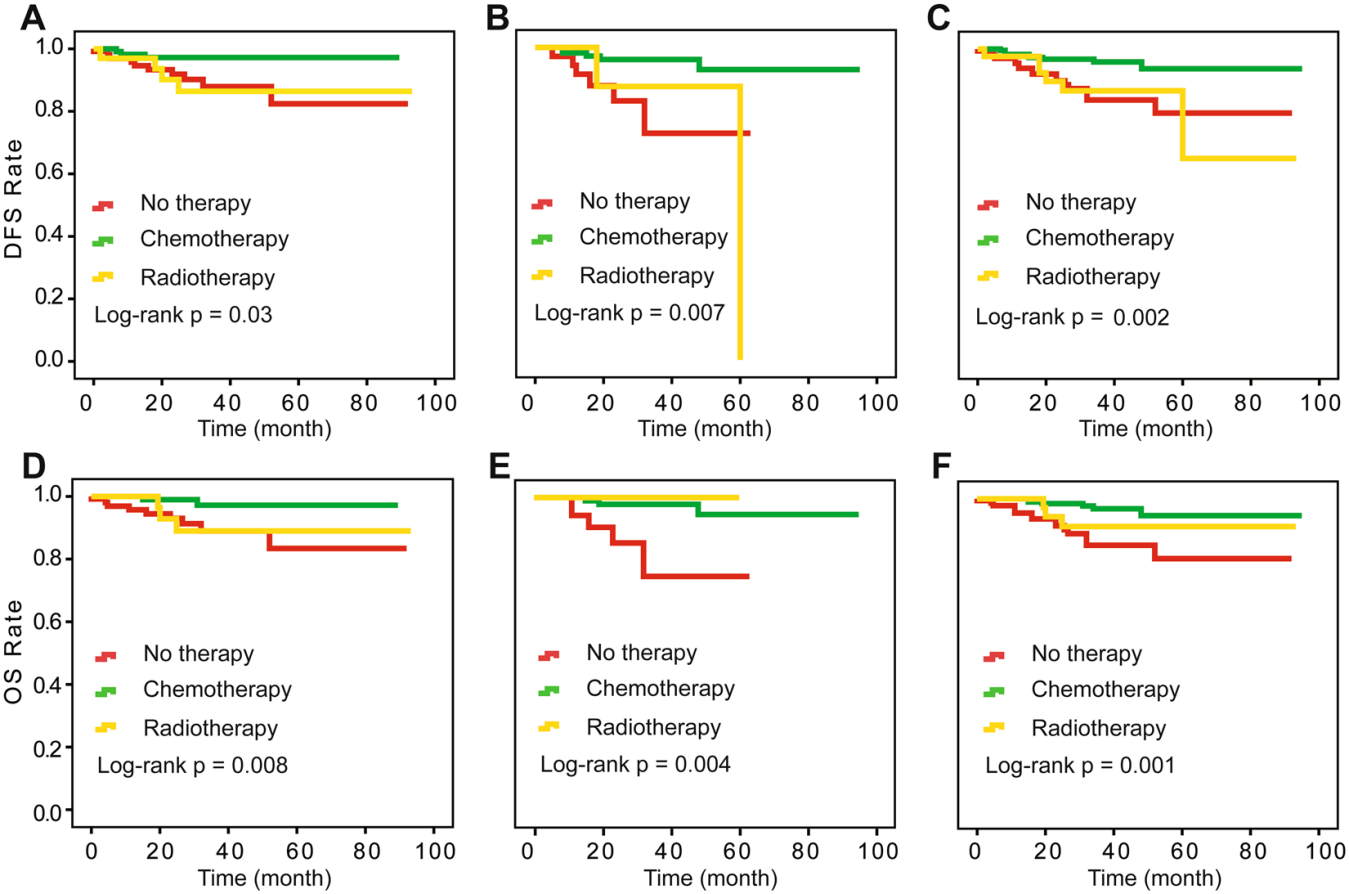
Kaplan-Meier survival estimates for patients with extra-cervical residual disease from the retrospective study, the prospective cohort and the two studies combined. Disease-free survival (DFS) curves of evaluated patients in the retrospective study (**A**), the prospective cohort (**B**) and the combined results (**C**). Overall survival (OS) curves of evaluated patients in the retrospective study (**D**), the prospective cohort (**E**) and the combined results (**F**).

**Figure 4:**
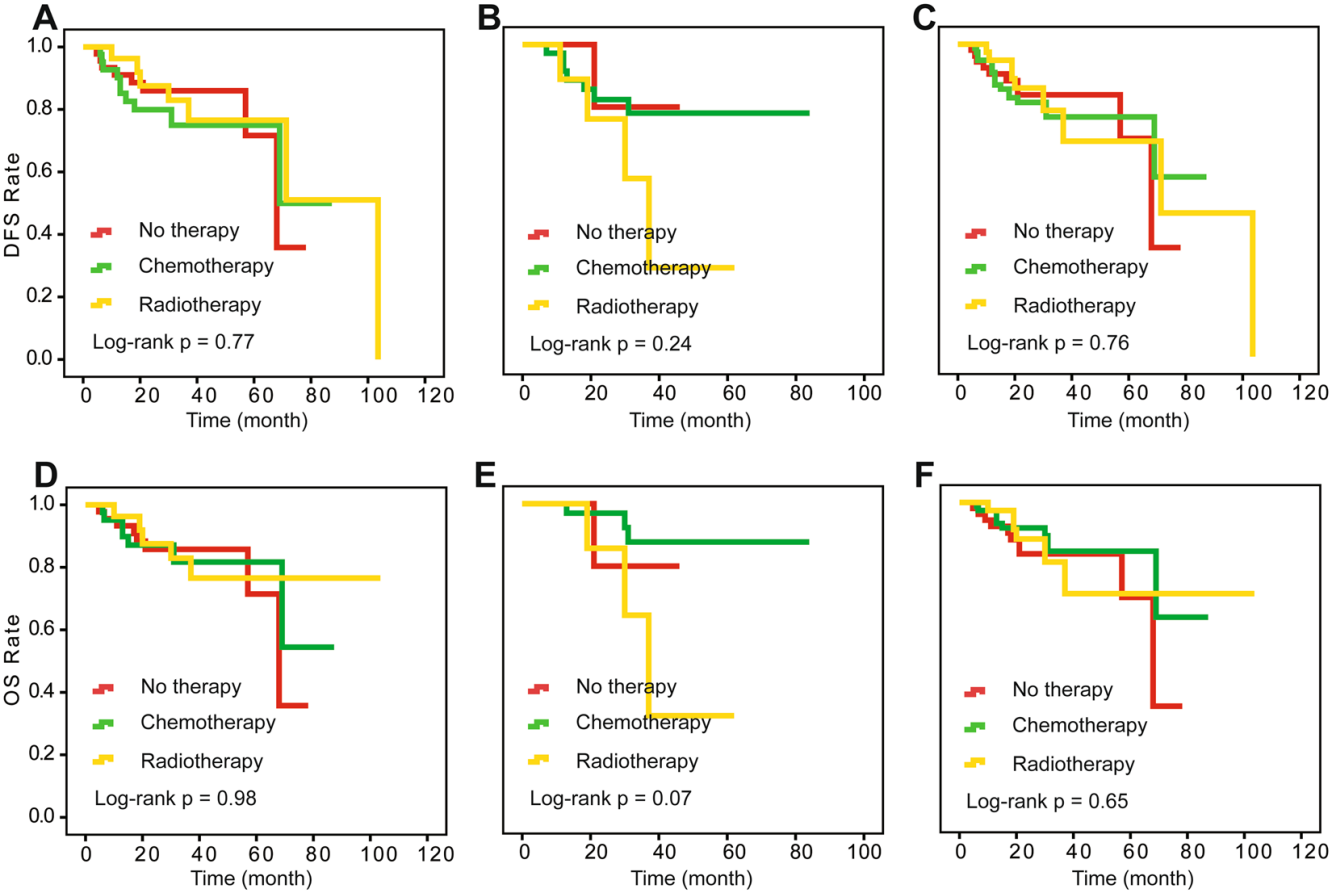
Kaplan-Meier survival estimates for patients with no response from the retrospective study, the prospective cohort and the two studies combined. Disease-free survival (DFS) curves of evaluated patients in the retrospective study (**A**), the prospective cohort (**B**) and the combined results (**C**). Overall survival (OS) curves of evaluated patients in the retrospective study (**D**), the prospective cohort (**E**) and the combined results (**F**).

We also investigated the postoperative treatment effect on survival at different levels of pathological response in surgical specimens using the Kaplan-Meier method. In the retrospective study, optimal response patients did not differ significantly in OS and DFS among adjuvant chemotherapy, radiotherapy and no therapy (log-rank P = 1.00, 1.00 for DFS and OS). For optimal response patients, even without therapy, the DFS and OS rates both reached over 90% (Fig. 1). For patients with intra-cervical residual disease patients from the retrospective study, the prospective cohort and the combination of the two studies, there was no statistical difference in outcomes among the three treatments (Fig. 2). For extra-cervical residual disease patients, survival differed among the three postoperative treatments (log-rank P = 0.03 and 0.008 for DFS and OS) (Fig. 3). These patients had the best clinical outcomes after receiving adjuvant chemotherapy, and these results were validated in the prospective study. The corresponding log-rank P-values were 0.90 and 0.92 for optimal response patients and 0.007 and 0.004 for extra-cervical residual disease patients for DFS and OS, respectively, in the prospective study. For patients with no response patients from the retrospective study, the prospective cohort and the combination of the two studies, there was no statistical difference in outcomes among the three treatments, too (Fig. 4).

**Figure 5:**
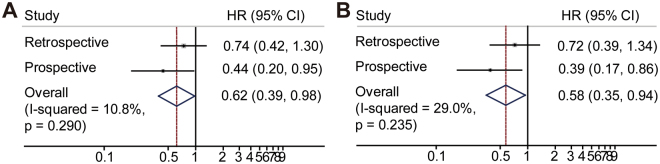
The combined univariate (**A**) and multivariate (**B**) DFS analysis results for adjuvant chemotherapy versus no therapy.

**Figure 6:**
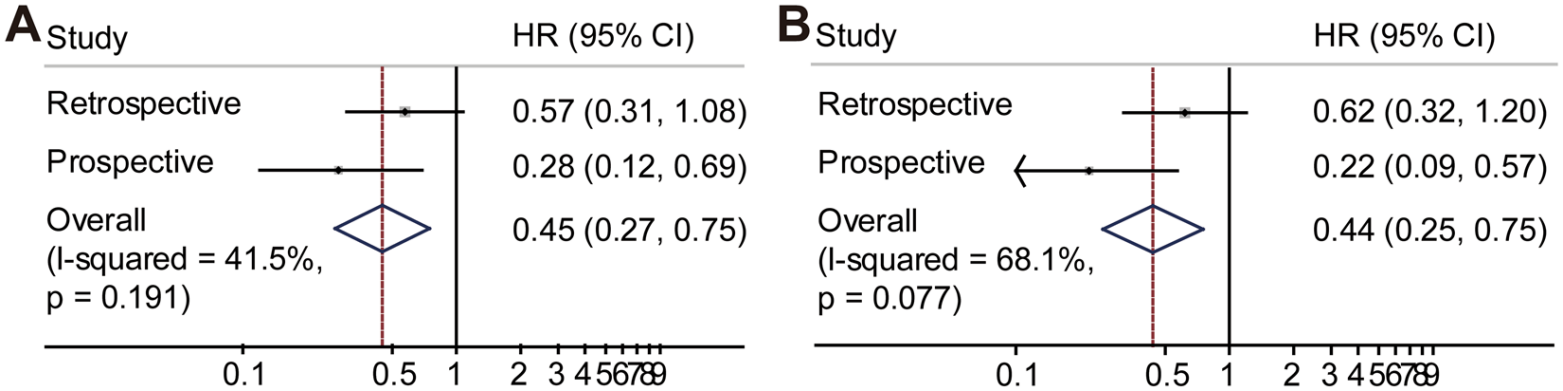
The combined univariate (**A**) and multivariate (**B**) OS analysis results for adjuvant chemotherapy versus no therapy.

We used joint analysis of the retrospective study and prospective cohort to analyze the postoperative treatment effect on survival. The results from the retrospective and prospective studies were combined. Univariate Cox analysis revealed the HRs for adjuvant chemotherapy and no therapy were 0.62 (95% CI, 0.39, 0.98, I2 = 10.8%) and 0.45 (95% CI, 0.27, 0.75, I2 = 41.5%) for DFS and OS, respectively. Multivariate Cox analysis revealed the HRs to be 0.58 (95% CI, 0.35, 0.94, I2 = 29%) and 0.44 (95% CI, 0.25, 0.75, I2 = 68.1%) for DFS (Fig. 5) and OS, respectively (Fig. 6).

Univariate and multivariate Cox regression for DFS and OS revealed that lymph node metastasis was the most important risk factor for survival. In the combined univariate analysis, the P values reached 0.000001 (HR = 3.26) and 0.00001 (HR = 3.42) for DFS and OS, and in the combined multivariate analysis, the P values were 0.002 and 0.00001 for DFS and OS, respectively (Supplementary Tables).

## Discussion

The treatment approach for FIGO stage IB2-IIB cervical cancer after surgery remains controversial. Adjuvant radiotherapy (including CCRT) is used in many countries, as recommended by the National Comprehensive Cancer Network (NCCN) clinical practice guidelines for cervical cancer with pathological risk factors following radical hysterectomy^16^. Gynecologic Oncology Group study 92 also found that radiotherapy after radical surgery significantly increased progression-free survival in patients with IB cervical cancer with negative and inter-medium-risk factors^17^. Postoperative radiotherapy with or without CCRT is recommended for patients with intermediate- or high-risk factors. Nobuhiro Takeshima *et al*. investigated the efficacy of chemotherapy alone as a postoperative adjuvant therapy for intermediate- and high-risk cervical cancer and found that adjuvant chemotherapy alone may help to treat patients with cervical cancer^10^. However, other studies found no statistical significance among adjuvant chemotherapy, radiotherapy and observation. Manfred Lahousen *et al*. concluded that adjuvant chemotherapy and radiation did not improve survival or recurrence rates in high-risk cervical cancer patients after radical hysterectomy, and the most important treatment for these patients appeared to be radical abdominal hysterectomy with systematic pelvic lymphadenectomy^18^.

NACT followed by radical hysterectomy has recently become an encouraging alternative therapeutic choice for patients with stage IB2-IIB cervical cancer. Some authors evaluated the possibility of adding adjuvant therapy for cervical cancer after NACT followed by radical surgery. Other studies concluded that adjuvant therapy yields promising results with a 5-year OS ranging from 72 to 83% with reduced extra-pelvic recurrences from adding adjuvant chemotherapy after radical surgery^19^. Huali Wang *et al*. studied adjuvant treatment for patients who accepted NACT followed by radical surgery and found that 179 of 256 patients received postoperative radiotherapy with a recurrence rate of 4.47%, which differed significantly from the 15.58% recurrence rate in those without radiotherapy (P = 0.000). Among 246 patients who needed further postoperative chemotherapy, 182 who received postoperative chemotherapy had a recurrence rate of 2.747%, while 64 patients who did not receive the chemotherapy had a recurrence rate of 10.93% (P = 0.007). These authors concluded that postoperative radiotherapy and chemo-adjuvant therapy both decreased the recurrence rate^20^. F. Landoni concluded that additional chemotherapy cycles benefitted patients with suboptimal response and intra-cervical residual disease, and both adjuvant chemotherapy and adjuvant radiation treatment did not improve the clinical outcome for patients with extra-cervical residual disease compared to those who received no further treatment^14^.

In our retrospective study, adjuvant chemotherapy supported OS in SCC patients compared with no therapy, which was also validated in a prospective cohort in which the advantage was more noticeable (P = 0.006, HR = 0.28) with a reduced recurrence rate (P = 0.04). Adjuvant radiotherapy did not significantly differ from no therapy for SCC patients in either study. In the combined univariate analysis, the HRs for adjuvant radiotherapy and no therapy were 0.91 (95% CI, 0.28, 0.89, I2 = 79.7%) and 1.21 (95% CI, 0.73, 2.00, I2 = 0.0%) for DFS and OS, respectively. In the Cox multivariate analysis, the HRs were 0.77 (95% CI, 0.46, 1.30, I2 = 0.0%) and 0.64 (95% CI, 0.37, 1.12, I2 = 0.0%), respectively, for DFS (Supplementary Figure 2) and OS (Supplementary Figure 3). For optimal response patients, no matter any treatments can obtain a very good clinical outcome. That is because that they mostly had no risk factors which we investigated in our previous study^21^. Intra-cervical residual disease patients had relatively worse survival and showed no differences among the three treatments (log-rank P = 0.82, 0.42, 0.69 for the DFS rate and 0.82, 0.59, 0.59 for the OS rate in the retrospective study, prospective cohort and combined analysis, respectively) (Fig. 2). Adjuvant chemotherapy showed a clear advantage on survival in extra-cervical residual disease patients compared to those without therapy. In addition, the clinical outcomes of the no-response patients were the worst. Adjuvant chemotherapy and adjuvant radiotherapy did not improve the DFS and OS rate compared with no therapy (log-rank P = 0.77, 0.24, 0.76 for the DFS rate and 0.98, 0.07, 0.65 for the OS rate in the retrospective study, prospective cohort and combined analysis, respectively) (Fig. 3).

Lymph node metastasis is the most important risk factor for cervical cancer patients^8,18,22,23^. Although it is not included in the current 2009 FIGO staging system, lymph node status is crucial for prognostically characterizing and managing cervical carcinoma. Kim *et al*. reported an association between the number of metastatic lymph nodes and the survival of patients treated with radical surgery compared to patients treated with NACT plus radical surgery^8^. In our study, lymph node metastasis was the most important independent risk factor for survival.

A strength of our study is that we performed the research with retrospective and prospective cohort studies with sufficiently large sample sizes.

In conclusion, we evaluated the correlation between clinical outcomes and postoperative treatments based on the pathological response for IB2-IIB2 cervical cancer patients with NACT followed by radical surgery. We found that patients who accepted adjuvant chemotherapy after surgery experienced beneficial clinical outcomes, especially those with extra-cervical residual disease. In addition, optimal response patients may not need further treatment.

## Materials and Methods

### Patients

This study included a retrospective cohort and a prospective cohort. The retrospective study data dated from 1999 to 2008. The prospective cohort began in 2002 until the present. The prospective cohort was performed at the Departments of Obstetrics and Gynecology at our institutions, and the registration number from Clinicaltrials.gov is NCT01628757. The inclusion criteria for the study included squamous cell carcinoma, age ≥18 and <70 years, accepted NACT followed by radical surgery and FIGO stage IB2-IIB. The study was conducted in accordance with approved guidelines. The clinical investigation followed the Declaration of Helsinki. All experimental protocols were approved by the Ethics Committee of Tongji Medical College at Huazhong University of Science and Technology. All eligible patients provided written informed consent before entering this study.

### Neoadjuvant chemotherapy

NACT used in our study was a cisplatin-based regimen, typically administered in 1–2 courses, depending on patient tolerance and response, and a few patients received an additional 1–2 cycles. The maximum duration was less than 2 months to avoid a delay in curative treatment.

### Pathological response

The pathological responses were retrospectively assessed as follows: PCR was defined as the complete disappearance of the tumor in the cervix and negative nodes; PR1 (partial response one) is defined as residual disease with less than 3 mm stromal invasion, including *in situ* carcinoma with or without lymphatic metastasis; and PR2 (partial response two) is defined a persistent residual disease with more than 3 mm stromal invasion in the surgical specimen. PR2 was divided into two parts: intra-cervical residual disease (persistent residual disease with >3 mm stromal invasion on the surgical specimen and negative nodes) and extra-cervical residual disease (persistent residual disease with >3 mm stromal invasion on the surgical specimen and positive nodes or positive parametria and/or surgical margins with negative nodes). We chose the 3-mm threshold as the lowest limit of an OPR (PCR + PR1) because it represents the maximal extension of FIGO stage IA1 cervical cancer. The histopathological diagnosis was confirmed by two pathologists per patient.

### Postoperative treatments

Adjuvant chemotherapy (cisplatin-based) after NACT followed by surgery was administered for 2 to 6 cycles per the same regimen as the NACT treatments. Adjuvant radiotherapy after NACT following surgery consisted of external beam irradiation and intra-cavity brachytherapy with or without concurrent chemotherapy (cisplatin, 30 mg/m2 every 6 weeks).

### Follow-up study

Patient follow-up was designed to be conducted every 3 months during the first year and every 6 months over the next four years after surgery. As per our database, follow-up was not performed for a few patients due to phone number changes. Efforts were made to contact them by their given address; however, we were still unable to connect with some patients, thus their data were excluded from the survival analysis.

### Statistical analysis

Survival was compared and estimated using the Kaplan-Meier method. The median follow-up time was calculated as the median observation time among all patients. The SPSS 13.0 software package was used to perform all statistical analyses. All reported P-values are two-sided, and we considered P-values less than 0.05 to be significant.

## Electronic supplementary material

Supplementary information
