# Supplementary material for: Adjuvant chemotherapy after surgery can improve clinical outcomes for patients with IB2-IIB cervical cancer with neoadjuvant chemotherapy followed by radical surgery
Source: Sci Rep. 2018 Apr 24;8:6443. doi: 10.1038/s41598-018-24413-z (PMC5915525; doi:10.1038/s41598-018-24413-z)
Supplement: Supplementary file 1 — Supplementary information [file 41598_2018_24413_MOESM1_ESM.pdf]

Adjuvant chemotherapy after surgery can improve clinical outcomes for patients with IB2-IIB cervical cancer with neoadjuvant chemotherapy followed by radical surgery

Haiying Sun[12#], Kecheng Huang[12#], Fangxu Tang[12], Xiong Li[12], Xiaoli Wang[12], Sixiang Long[12], Shasha Zhou[12], Suolangquzhen[12], Jianwei Zhang[12], Ruoqi Ning[12], Shuang Li[12], Shixuan Wang[12*] , Ding Ma[12*]

Supplementary figure 1. Patients' flowchart in the prospective study.

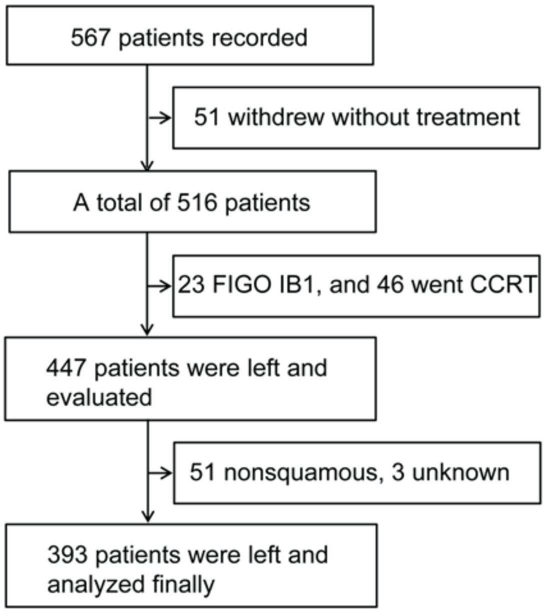

A total of 516 patients were included in this study in the beginning; 23 patients with FIGO IB1 stage cervical cancer were excluded from further analysis; 493 patients with FIGO stage IB2-IIB were left and their data were finally analyzed.

Supplementary figure 2. High-risk prognostic factors' distribution among the responders and non-responders.

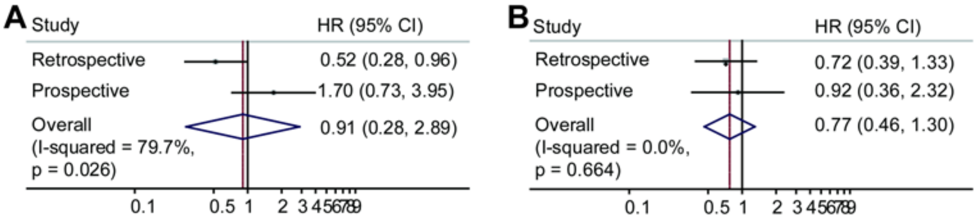

The ratio of cases with (A) positive vaginal surgical margin (P = 0.002), (B) positive parametrial infiltration (P < 0.001) or (C) positive lymph node (P < 0.001) were significantly high in the non-responder group: (A) P = 0.002; (B) P < 0.001; (C) P < 0.001.

Supplementary figure 3. Comparability of post-operative treatments among responders.

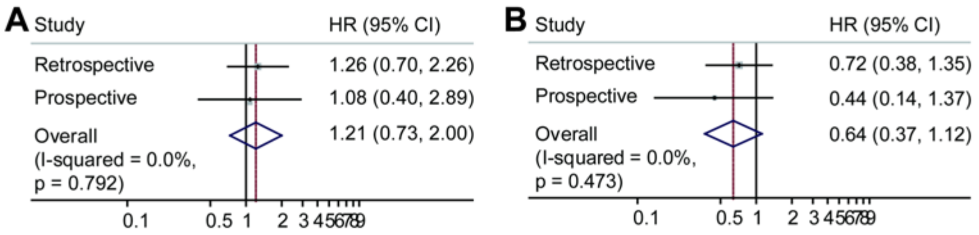

Chemotherapy led to significantly higher disease-free survival rate than any other therapy with $P < 0.001$. CCRT, concurrent chemoradiotherapy.

Supplementary Table 1. Univariate Cox regression for DFS in the retrospective study.

**Variables**	HR	95% CI	*P*
**Postoperative treatment**			
Chemotherapy vs No therapy	0.74	0.42 to 1.3	0.30
Raditherapy vs No therapy	0.52	0.28 to 0.96	0.04
**Age**			
>44 vs ≤44 years	1.57	0.97 to 2.53	0.07
**FIGO stage**			
IIA vs IB2	2.48	1.15 to 5.35	0.02
IIB2 vs IB2	2.54	1.23 to 5.28	0.01
**Pathological response**			
Intra-cervical residual disease vs Optimal	15866.87	6.44E-47 to 3.91E+54	0.87
Extra-cervical residual disease vs Optimal	47531.36	1.93E-46 to 1.17E+55	0.86
No response vs Optimal	42355.92	1.72E-46 to 1.04E+55	0.86
**Grade**			
G2 vs G1	2.32	0.55 to 9.71	0.25
G3 vs G1	3.83	0.91 to 16.09	0.07
Undetermined vs G1	1.90	0.42 to 8.48	0.40
**Tumor size**			
>4cm vs ≤4cm	1.30	0.77 to 2.17	0.32
**Parametrial infiltration**			
Positive vs Negative	2.50	1.36 to 4.61	0.00
**Vaginal surgical margin**			
Positive vs Negative	2.21	1.05 to 4.61	0.04
**Lymph node metastasis**			
Positive vs Negative	3.54	2.19 to 5.72	0.00

Supplementary Table 2. Univariate Cox regression for OS in the retrospective study.

**Variables**	HR	95% CI	*P*
**Postoperative treatment**			
Chemotherapy vs No therapy	0.57	0.31 to 1.08	0.08
Raditherapy vs No therapy	1.26	0.70 to 2.26	0.44
**Age**			
>44 vs ≤44 years	1.60	0.97 to 2.66	0.07
**FIGO stage**			
IIA vs IB2	2.74	1.16 to 6.46	0.02
IIB2 vs IB2	3.05	1.36 to 6.88	0.007
**Pathological response**			
Intra-cervical residual disease vs Overall optimal	15224.79	6.04E-51 to 3.84E+58	0.88
Extra-cervical residual disease vs Overall optimal	53843.44	2.14E-50 to 1.36E+59	0.86
No response vs Overall optimal	40615.29	1.61E-50 to 1.02E+59	0.87
**Grade**			
G2 vs G1	4.24	0.57 to 31.24	0.16
G3 vs G1	6.91	0.93 to 51.11	0.06
Undetermined vs G1	3.82	0.50 to 29.37	0.2
**Tumor size**			
>4cm vs ≤4cm	1.12	0.66 to 1.93	0.67
**Parametrial infiltration**			
Positive vs Negative	2.82	1.52 to 5.24	0.001
**Vaginal surgical margin**			
Positive vs Negative	2.46	1.17 to 5.17	0.02
**Lymph node metastasis**			
Positive vs Negative	3.64	2.21 to 5.99	4.08E-07

Supplementary Table 3. Multivariate Cox regression for DFS in the retrospective study.

**Variables**	HR	95% CI	*P*
**Postoperative treatment**			
Chemotherapy vs no therapy	0.72	0.39 to 1.34	0.3
Raditherapy vs no therapy	0.72	0.39 to 1.33	0.29
**FIGO stage**			
IIA vs IB2	2.57	1.13 to 5.83	0.02
IIB2 vs IB2	2.15	0.98 to 4.72	0.06
**Pathological response**			
Intra-cervical residual disease vs Overall optimal	22579.92463	9.28E-60 to 5.50E+67	0.89
Extra-cervical residual disease vs Overall optimal	31320.11077	1.29E-59 to 7.63E+67	0.89
No response vs Overall optimal	42995.39854	1.77E-59 to 1.05E+68	0.89
**Parametrial infiltration**			
Positive vs Negative	1.60	0.78 to 3.30	0.2
**Vaginal surgical margin**			
Positive vs Negative	1.17	0.51 to 2.67	0.72
**Lymph node metastasis**			
Positive vs Negative	2.55	1.31 to 4.98	0.006

Supplementary Table 4. Multivariate Cox regression for OS in the retrospective study.

**Variables**	HR	95% CI	*P*
**Postoperative treatment**			
Chemotherapy vs no therapy	0.62	0.32 to 1.20	0.16
Raditherapy vs no therapy	0.72	0.38 to 1.35	0.31
**FIGO stage**			
IIA vs IB2	2.97	1.18 to 7.48	0.02
IIB2 vs IB2	2.76	1.14 to 6.68	0.02
**Pathological response**			
Intra-cervical residual disease vs Overall optimal	8710.64	7.03E-38 to 1.08E+45	0.85
Extra-cervical residual disease vs Overall optimal	12489.27	1.01E-37 to 1.55E+45	0.85
No response vs Overall optimal	13900.93	1.12E-37 to 1.72E+45	0.84
**Parametrial infiltration**			
Positive vs Negative	1.56	0.75 to 3.24	0.23
**Vaginal surgical margin**			
Positive vs Negative	1.34	0.59 to 3.09	0.49
**Lymph node metastasis**			
Positive vs Negative	2.55	1.27 to 5.14	0.009

Supplementary Table 5. Univariate Cox regression for DFS in the prospective study.

**Variables**	HR	95% CI	***P***
**Postoperative treatment**			
Chemotherapy vs No therapy	0.44	0.2 to 0.95	0.04
Raditherapy vs No therapy	1.7	0.73 to 3.95	0.22
**Age**			
>44 vs ≤44 years	1.69	0.89 to 3.19	0.11
**FIGO stage**			
IIA vs IB2	2.1	0.69 to 6.42	0.2
IIB2 vs IB2	3.03	1.17 to 7.88	0.02
**Pathological response**			
Intra-cervical residual disease vs Overall optimal	1.36	0.31 to 6.06	0.68
Extra-cervical residual disease vs Overall optimal	4.65	1.05 to 20.72	0.04
No response vs Overall optimal	4.01	0.89 to 18	0.07
**Grade**			
G2 vs G1	2.18	0.23 to 21	0.5
G3 vs G1	2.67	0.36 to 19.93	0.34
Undetermined vs G1	3.95	0.52 to 29.82	0.18
**Tumor size**			
>4cm vs ≤4cm	0.79	0.4 to 1.56	0.5
**Parametrial infiltration**			
Positive vs Negative	1.59	0.48 to 5.27	0.45
**Vaginal surgical margin**			
Positive vs Negative	4.53	1.88 to 10.93	0.001
**Lymph node metastasis**			
Positive vs Negative	2.86	1.52 to 5.39	0.001

Supplementary Table 6. Univariate Cox regression for OS in the prospective study.

**Variables**	HR	95% CI	*P*
**Postoperative treatment**			
Chemotherapy vs No therapy	0.28	0.12 to 0.69	0.006
Raditherapy vs No therapy	1.08	0.4 to 2.89	0.88
**Age**			
>44 vs ≤44 years	1.89	0.87 to 4.14	0.11
**FIGO stage**			
IIA vs IB2	1.38	0.28 to 6.86	0.69
IIB2 vs IB2	3.97	1.18 to 13.31	0.03
**Pathological response**			
Intra-cervical residual disease vs Overall optimal	0.79	0.17 to 3.71	0.76
Extra-cervical residual disease vs Overall optimal	3.29	0.72 to 15.02	0.13
No response vs Overall optimal	2.19	0.45 to 10.53	0.33
**Grade**			
G2 vs G1	0.67	0.04 to 10.79	0.78
G3 vs G1	1.71	0.22 to 13.11	0.6
Undetermined vs G1	3	0.39 to 23.04	0.29
**Tumor size**			
>4cm vs ≤4cm	0.6	0.27 to 1.36	0.22
**Parametrial infiltration**			
Positive vs Negative	2.1	0.65 to 6.76	0.21
**Vaginal surgical margin**			
Positive vs Negative	6.71	2.53 to 17.81	0.0001
**Lymph node metastasis**			
Positive vs Negative	2.82	1.31 to 6.07	0.008

Supplementary Table 7. Multivariate Cox regression for DFS in the prospective study.

**Variables**	HR	95% CI	***P***
**Postoperative treatment**			
Chemotherapy vs No therapy	0.39	0.17 to 0.86	0.02
Raditherapy vs No therapy	0.92	0.36 to 2.32	0.86
**Vaginal surgical margin**			
Positive vs Negative	3.06	1.21 to 7.77	0.02
**Lymph node metastasis**			
Positive vs Negative	2.56	1.3 to 5.06	0.007

Supplementary Table 8. Multivariate Cox regression for OS in the prospective study.

**Variables**	HR	95% CI	*P*
**Postoperative treatment**			
Chemotherapy vs No therapy	0.22	0.088 to 0.57	0.002
Raditherapy vs No therapy	0.445	0.14 to 1.37	0.16
**Vaginal surgical margin**			
Positive vs Negative	5.53	1.89 to 16.15	0.002
**Lymph node metastasis**			
Positive vs Negative	2.898	1.27 to 6.59	0.01

Supplementary Table 9. Univariate Cox regression for DFS in the combined analysis.

**Variables**	HR	95% CI	***P***
**Postoperative treatment**			
Chemotherapy vs No therapy	0.62	0.39 to 0.97	0.04
Raditherapy vs No therapy	1.49	0.94 to 2.37	0.09
**Age**			
>44 vs ≤44 years	1.61	1.10 to 2.36	0.02
**FIGO stage**			
IIA vs IB2	2.36	1.26 to 4.43	0.007
IIB2 vs IB2	2.72	1.53 to 4.86	0.001
**Pathological response**			
Intra-cervical residual disease vs Overall optimal	3.63	0.87 to 15.20	0.08
Extra-cervical residual disease vs Overall optimal	11.28	2.73 to 46.70	0.001
No response vs Overall optimal	9.95	2.40 to 41.38	0.002
**Grade**			
G2 vs G1	1.65	0.66 to 4.14	0.29
G3 vs G1	2.60	1.03 to 6.57	0.04
Undetermined vs G1	1.32	0.47 to 3.76	0.6
**Tumor size**			
>4cm vs ≤4cm	1.10	0.73 to 1.66	0.64
**Parametrial infiltration**			
Positive vs Negative	2.21	1.31 to 3.72	0.003
**Vaginal surgical margin**			
Positive vs Negative	2.82	1.61 to 4.96	0.0003
**Lymph node metastasis**			
Positive vs Negative	3.26	2.23 to 4.77	0.000001

Supplementary Table 10. Univariate Cox regression for OS in the combined analysis.

**Variables**	HR	95% CI	***P***
**Postoperative treatment**			
Chemotherapy vs No therapy	0.45	0.27 to 0.75	0.002
Raditherapy vs No therapy	1.23	0.74 to 2.03	0.42
**Age**			
>44 vs ≤44 years	1.70	1.11 to 2.60	0.01
**FIGO stage**			
IIA vs IB2	2.46	1.17 to 5.14	0.02
IIB2 vs IB2	3.29	1.68 to 6.45	0.001
**Pathological response**			
Intra-cervical residual disease vs Overall optimal	2.63	0.62 to 11.15	0.19
Extra-cervical residual disease vs Overall optimal	9.98	2.41 to 41.41	0.002
No response vs Overall optimal	7.30	1.74 to 30.73	0.007
**Grade**			
G2 vs G1	3.42	0.83 to 14.17	0.09
G3 vs G1	5.68	1.37 to 23.58	0.02
Undetermined vs G1	3.43	0.77 to 15.32	0.11
**Tumor size**			
>4cm vs ≤4cm	0.91	0.58 to 1.43	0.69
**Parametrial infiltration**			
Positive vs Negative	2.73	1.62 to 4.61	0.0002
**Vaginal surgical margin**			
Positive vs Negative	3.33	1.84 to 6.00	0.00007
**Lymph node metastasis**			
Positive vs Negative	3.42	2.26 to 5.18	0.000001

Supplementary Table 11. Multivariate Cox regression for DFS in the combined analysis.

**Variables**	HR	95% CI	***P***
**Postoperative treatment**			0.07
Chemotherapy vs No therapy	0.58	0.36 to 0.93	0.02
Raditherapy vs No therapy	0.70	0.41 to 1.17	0.17
**Age**			
>44 vs ≤44 years	1.73	1.14 to 2.61	0.01
**FIGO stage**			0.006
IIA vs IB2	2.76	1.36 to 5.58	0.005
IIB2 vs IB2	2.87	1.49 to 5.53	0.002
**Pathological response**			0.045
Intra-cervical residual disease vs Overall optimal	3.51	0.82 to 14.96	0.09
Extra-cervical residual disease vs Overall optimal	5.55	1.23 to 25.04	0.03
No response vs Overall optimal	6.36	1.46 to 27.75	0.01
**Grade**			0.006
G2 vs G1	2.12	0.83 to 5.41	0.12
G3 vs G1	3.81	1.46 to 9.94	0.006
Undetermined vs G1	1.74	0.61 to 4.97	0.3
**Lymph node metastasis**			
Positive vs Negative	2.48	1.42 to 4.35	0.002

Supplementary Table 12. Multivariate Cox regression for OS in the combined analysis.

**Variables**	HR	95% CI	***P***
**Postoperative treatment**			0.004
Chemotherapy vs No therapy	0.41	0.24 to 0.70	0.001
Raditherapy vs No therapy	0.62	0.35 to 1.10	0.1
**Age**			
>44 vs ≤44 years	1.87	1.18 to 2.97	0.008
**FIGO stage**			0.003
IIA vs IB2	3.00	1.27 to 7.09	0.01
IIB2 vs IB2	3.95	1.77 to 8.80	0.001
**Grade**			0.001
G2 vs G1	5.43	1.30 to 22.79	0.02
G3 vs G1	10.51	2.47 to 44.70	0.001
Undetermined vs G1	4.62	1.03 to 20.78	0.046
**Lymph node metastasis**			
Positive vs Negative	4.23	2.67 to 6.70	0.000001
